# Causal Responsibility Division of Chronological Continuous Treatment Based on Change-Point Detection

**DOI:** 10.3390/e25081164

**Published:** 2023-08-03

**Authors:** Hang Liu, Tiefeng Ma, Conan Liu, Shuangzhe Liu

**Affiliations:** 1 School of Statistics, Southwestern University of Finance and Economics, Chengdu 611130, China; gwyzmz@outlook.com (H.L.); matiefeng@swufe.edu.cn (T.M.); 2 Business School, University of New South Wales, Sydney, NSW 2052, Australia; conan.liu88@gmail.com; 3 Faculty of Science and Technology, University of Canberra, Bruce, ACT 2617, Australia

**Keywords:** causal inference, change-point detection, harmonic responsibility division, propensity score

## Abstract

This paper introduces a novel approach, called causal relation quantification, based on change-point detection to address the issue of harmonic responsibility division in power systems. The proposed method focuses on determining the causal effect of chronological continuous treatment, enabling the identification of crucial treatment intervals. Within each interval, three propensity-score-based algorithms are executed to assess their respective causal effects. By integrating the results from each interval, the overall causal effect of a chronological continuous treatment variable can be calculated. This calculated overall causal effect represents the causal responsibility of each harmonic customer. The effectiveness of the proposed method is evaluated through a simulation study and demonstrated in an empirical harmonic application. The results of the simulation study indicate that our method provides accurate and robust estimates, while the calculated results in the harmonic application align closely with the real-world scenario as verified by on-site investigations.

## 1. Introduction

Causal inference considers the assumptions, study designs, and estimation strategies that allow researchers to draw causal conclusions based on data. It is increasingly used in social, medical, and physical sciences. However, most studies in these areas concentrate on binary or categorical treatments [1,2,3]. For areas whose treatment has both chronological order and continuous value, such as the harmonic responsibility division in a power system, the  current literature is relatively sparse.

In the field of causal inference, the Potential Outcome Framework, specifically the Rubin Causal Model (RCM) [4], provides a common framework for studying causality in observational data. Propensity score (PS) methods have gained popularity in causal inference [5,6,7]. However, these methods primarily focus on binary treatments. While generalized propensity score (GPS) methods [8,9] have been proposed and extended to handle categorical or continuous treatments, they are still limited in addressing causal inference for chronological continuous treatment.

When considering time series data as the treatment variable, recent studies in causal inference can be classified into two types based on different time settings. One type involves a time-invariant treatment effect [10,11,12], while the other type involves a time-varying treatment effect [13]. However, existing models predominantly focus on treatments that have dichotomous values, regardless of whether the treatment is time-invariant or time-varying. This limitation indicates that these models are primarily applicable to binary treatment or chronological binary treatment scenarios. In the context of the responsibility division problem, where the treatment varies over time and assumes continuous values at each time point, the existing approaches are unable to directly address this challenge. Hence, there is a need to develop a novel method capable of generating causal effect estimations specifically for a chronological continuous treatment.

Our motivation stems from the harmonic responsibility division problem, which involves identifying and estimating the impact of customers generating harmonic interference on the power system, as formalized in model (1). Due to the chronological order of the data, ordinary regression methods may not be suitable. Hence, we approach the problem from a causal perspective by estimating the causal effects of the chronological continuous treatment.

Given the practical application demand and the scarcity of methods for estimating the effect of a chronological continuous treatment, we propose a new method called causal relation quantification based on change-point detection (CRQ-CPD).

The proposed method comprises two main components: (1) identifying critical treatment intervals and (2) computing the causal effect based on the identified intervals. Throughout the entire treatment sequence, observations carry different amounts of information, with intervals exhibiting significant fluctuations containing more informative data than stable intervals. To identify treatment intervals with substantial fluctuations, our method employs change-point detection techniques. Leveraging the inherent properties of change points, we divide each identified treatment interval into control and trial groups. Subsequently, using three classical propensity-score-based algorithms, we calculate the causal effects for each interval and integrate them to determine the overall causal effect of the entire chronological continuous treatment sequence, which is regarded as the harmonic responsibility.

The contributions of this paper can be summarized as follows:

(1) **Proposal of a New Method**: This paper introduces a novel data-driven method that leverages the concept that observations contain distinct information. By utilizing change-point detection techniques, the proposed method identifies crucial intervals and calculates the causal effect of a chronological continuous treatment. This method extends the existing treatment types (binary, categorical, continuous, and chronological binary) to include chronological continuous treatment. Moreover, by utilizing partial data instead of the entire sequence, the proposed method enhances computational efficiency and improves the conversion rate of data information.
(2) **Solving the Harmonic Responsibility Division Problem**: Building upon the proposed method for estimating the causal effect of a chronological continuous treatment, this paper offers a new perspective on the division of harmonic responsibility by adopting a data-driven methodology rather than constructing physical models. The proposed causality-based approach is more robust and less susceptible to the influence of undetected customers, thereby offering a more reliable solution to the problem.

The remainder of this paper is organized as follows: Section 2 provides information on the practical problem of harmonic responsibility division and introduces the corresponding model. Section 3 describes the proposed methodology in detail. Section 4 evaluates the performance of the new approach in a simulation study and using real-world harmonic pollution data. Section 5 presents our concluding remarks on the study.

## 2. Problem Description and Model Setting

Harmonic pollution poses a significant challenge in power grids, as it directly leads to voltage distortion, decreased transformer capacity, and accelerated aging of equipment. Therefore, accurately estimating the responsibility of each customer in generating harmonics is crucial to effectively mitigate harmonic pollution.

Existing methods for harmonic responsibility division primarily rely on harmonic voltage projection, which is calculating the percentage of projection caused by individual harmonic sources at the Point of Common Coupling (PCC) [14,15,16]. However, these methods have two limitations. Firstly, they fail to allocate responsibility accurately among end customers within a feeder line that has multiple branches and serves multiple users. Secondly, they require the construction of physical models, which demands specific data that are not always available in power-quality monitoring systems. To address these shortcomings, a new method solely based on monitoring data is needed to allocate harmonic responsibilities among multiple customers without relying on physical models.

**Figure 1:**
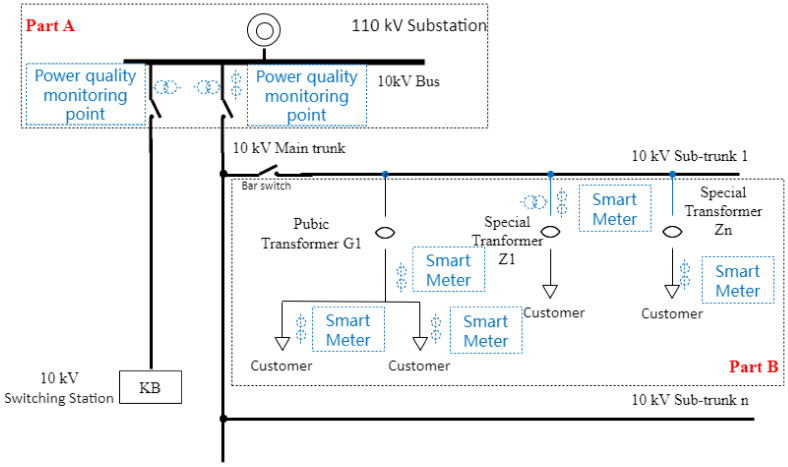
Schematic diagram of monitoring points in power quality monitoring system and electricity information collection system.

The most direct method is to set up a harmonic monitoring device for each probable harmonic customer. However, this is extremely costly. To efficiently reduce costs, the harmonic monitoring equipment is installed only at the main trunk to obtain the information of overall harmonic voltage, while the data of each customer can be collected from their household smart meter, which provides information on their individual power consumption behavior. Figure 1 illustrates this setup, where statistical data on overall harmonic voltages at PCC can be collected at Part A, and the data of average active power can be collected at Part B. The monitoring data used in the subsequent analysis include the harmonic voltage at the Point of Common Coupling (PCC), which represents the overall harmonic levels, as well as the average active power data, which reflect the power consumption of each customer.

Based on the collected monitoring data, the harmonic responsibility division problem can be formalized as

(1) $$Y_{t}=\beta_{0}+\beta_{1}X_{1t}+\beta_{2}X_{2t}+\cdots +\beta_{K}X_{Kt}+\epsilon_{t},$$

where $Y_{t}$ is the overall harmonic levels at time point *t*, $X_{1t},\cdots ,X_{Kt}$ are the customers’ power consumption at time point *t*, *K* is the number of customers, $\beta_{K}$ is the effect of the *K*th customer on the harmonic level, and $\epsilon_{t}$ is the disturbance at time point *t*.

In the model (1), *Y* represents the harmonic voltage at the PCC obtained from the power quality monitoring system, while *X* denotes the customers’ average active power data collected from the electricity information collection system. The current device record is relatively accurate, so the model does not consider the errors in the variables.

Derived from the practical harmonic responsibility division problem, the model (1) exhibits three key characteristics:

(1) *X* and *Y* all vary over time and have continuous values at each time point; in short, they are chronological continuous variables.
(2) *X* exhibits change points over time. *X* represents customers’ power consumption behavior, which can vary over time due to factors such as the nature of their business. For instance, a solar user which needs to convert the current generated by solar panels into the alternating current used by household appliances may exhibit distinct behavioral patterns during daylight hours compared with nighttime. These behavioral changes manifest as change points in the data.
(3) The β is assumed to remain constant throughout the entire time period. The β of each customer is regarded as its harmonic responsibility. Because the property of a certain customer’s appliance is fixed, and this property affects the harmonic emission levels of a customer, we assume that the β is fixed over time.

Based on these characteristics, direct utilization of regression methods, like ordinary least square (OLS), is not well-suited for analyzing the responsibility division problem. Therefore, from a causality perspective, we propose a novel CRQ-CPD method to estimate the β by identifying crucial intervals and estimating the causal effect of chronological continuous treatment to solve the harmonic responsibility division problem.

## 3. Causal Relation Quantification Based on Change-Point Detection (CRQ-CPD)

This section describes the details of the CRQ-CPD method. This method comprises two main steps:

- **Identification of crucial treatment intervals**: This step utilizes a change-point detection technique to identify treatment intervals where observations exhibit large fluctuations;
- **Treatment effect calculation based on the identified intervals**: In this step, three classical PS-based algorithms are used to calculate the causal effects for each interval. These effects are then integrated to obtain the overall causal effect of the chronological continuous treatment.

The obtained overall causal effects are the calculated harmonic responsibility of customers, i.e., the estimator of β.

The estimation process consists of two steps. In the first step, we identify informative data points and data intervals by utilizing change-point detection. This step solely requires the assumption of the existence of change points in the variable *X* and does not rely on the assumption of normality. In the second step, we divide the data within each interval into control and trial groups based on the nature of the change points. We then employ three classical PS-based methods to estimate the causal effect of *X* on *Y* within these intervals. During this step, Rubin’s three key assumptions are necessary for the three PS-based methods, but there is no requirement for the assumption of normality in the data distribution.

For the sake of subsequent instructions, we begin with notions before delving into the detailed method steps: Without loss of generality, the customer $X_{k}$ is shown as an example to illustrate the process of its harmonic responsibility estimation. In this case, the $X_{k}$ is denoted as the treatment variable *A*, while the remaining customers $X_{-k}$ are denoted as covariates *Z*:

$$\begin{array}{cc} Z & =(Z_{1},Z_{2},\cdots ,Z_{K-1})\\ \; & ≜X_{-k}=\left(X_{1},\cdots ,X_{k-1},X_{k+1},\cdots ,X_{K}\right). \end{array}$$

### 3.1. Identification of Crucial Treatment Intervals

In this step, we identify the critical treatment intervals of *A* based on the change points. In Section 2, we mentioned that model (1) requires the presence of change points in the customer variables, which manifest as distinct changes in the customers’ power consumption behavior in practical scenarios. Here, we provide information on change points in statistics.

Change points refer to the points in time where the distribution of a series of data suddenly behaves differently [17]. For the chronological continuous treatment sequence $A_{t}(t=1,\cdots ,N$, *N* is the length of the sequence), if there exists a time τ(1<τ<N) at which the distribution between the subsequences ($A_{1},\cdots ,A_{\tau }$) and ($A_{\tau +1},\cdots ,A_{N}$) differs significantly, then τ is identified as a change point. In the case of multiple such points, multiple change points have occurred.

Suppose there are *J* change points in *A*. Centered on a certain change point $\tau_{j}$(j=1,⋯,J), extract *h* data points around this change point as the *j*th treatment interval; *h* is the bandwidth used in the change-point detection.

**Definition** **1.** **(*Treatment interval*)**. *For any change point $\tau_{j}$ of the treatment sequence $A_{t}\left(t=1,\cdots ,N\right)$, the time index set $T_{j}=\left[\tau_{j}-\frac{h}{2},\tau_{j}+\frac{h}{2}\right]$ is the jth treatment interval.*

Within the *j*th treatment interval, data points of the *A* located on the same side (left or right) of the change point $\tau_{j}$ follow the same distribution, and distributions of data points differ between opposing sides. Based on the different distributions, points located on the left side of the change point can be regarded as in the *control group*, and those on the right side as in the *trial group*, shown as

$$\begin{array}{c} control\;group:\;T_{j}^{0}=\left[\tau_{j}-\frac{h}{2},\tau_{j}\right],\\ trial\;group:\;T_{j}^{1}=\left[\tau_{j}+1,\tau_{j}+\frac{h}{2}\right]. \end{array}$$

If there is no significant change in covariates *Z* in this region, then the change in *Y* can be regarded as the causal effect of *A* within this treatment interval.

**Figure 2:**
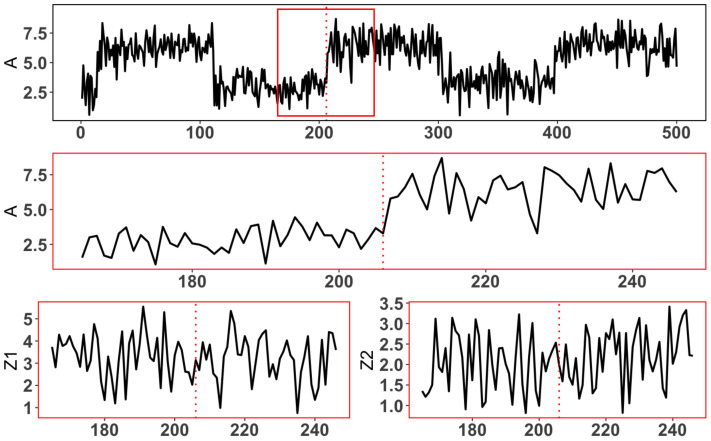
The top plot is the whole chronological continuous treatment variable *A*, the middle plot is the observations of *A* in selected treatment interval around a change point (the magnified image of the sequence in the red rectangle of the **top** plot), and the **bottom** plots are the corresponding interval of two covariates, with red dotted lines indicating locations of change points.

Change-point detection provides several advantages in calculating causal effect:

(1) **Information-rich intervals**: As discussed in Section 1, the observations within the identified intervals exhibit larger variations, indicating that they contain more valuable information. By focusing on these intervals, we can capture the main features of the treatment variable and make more efficient use of the available data.
(2) **Preservation of chronological order**: The partitioning of data into control and trial groups based on the identified intervals preserves the chronological order of the treatment to some extent. This means that the order of treatment times, rather than the treatment values themselves, is used to segment the intervals. Consequently, it becomes impossible to assign points to the wrong group (points in the trial group would not be mistakenly assigned to the control group and vice versa).
(3) **Covariate balance control**: Within each treatment interval, as illustrated in Figure 2, the treatment variable *A* of the points exhibits apparent fluctuations, while the other covariates *Z* remain relatively stable. This feature facilitates easier control of covariate balance and enables the accurate estimation of treatment effects after the interval identification process.

### 3.2. Treatment Effect Calculation Based on the Identified Intervals

Following the contents of Section 3.1, the *J* crucial intervals are identified according to the *J* change points of *A*. This step first shows the specific calculation process of the causal effect within each treatment interval, then demonstrates how these effects are subsequently integrated to derive the overall causal effect of the entire chronological continuous treatment sequence.

#### 3.2.1. Causal Effect within Treatment Intervals

To obtain the causal effect within the *j*th treatment interval, we first extract the corresponding data of covariates *Z* and outcome variable *Y* and divide them into a control group with time index $t\in T_{j}^{0}$ and trial group with time index $t\in T_{j}^{1}$. Then, we employ three propensity-score-based techniques, including reweighting (inverse probability weighting, IPW) [18], matching [19], and stratification [20], to compute the causal effects of this interval.

The key point of IPW is to assign weights based on the propensity score to each unit and compute the difference in the weighted outcomes between control and trial groups. The matching method identifies the matched units between two groups and measures the outcome’s difference between these units. The stratification method stratifies the units into several strata based on the propensity score and directly compares only trial and control units that fall into the same strata.

The estimation of causal effect within the *j*th treatment interval with the above three methods is shown as Equations (2), (4) and (7).

- **IPW estimator:**
(2) $${\hat{ATE}}_{IPW}^{j}=\frac{1}{h}\left(\sum_{t\in T_{j}^{1}}\frac{Y_{t}}{e(Z_{t})}-\sum_{t\in T_{j}^{0}}\frac{Y_{t}}{1-e(Z_{t})}\right).$$
  $e(Z_{t})$ in Equation (2) is the propensity score, defined as the conditional probability of the unit *t* being in trial group given the covariates $Z_{t}$, regardless of the actual position of unit *t* in (the control or trial group), shown as
  (3) $$e\left(Z_{t}\right)=Pr\left(G_{t}=1|Z_{t}\right),$$
  where $G_{t}=I\left(t\in T_{j}^{1}\right)$ is an indicator variable that represents whether unit *t* belongs to the trial group $T_{j}^{1}$.
- **Matching estimator:**
  (4) $${\hat{ATE}}_{Mat}^{j}=\frac{1}{h}\sum_{t\in T_{j}}\left({\hat{Y}}_{t}\left(1\right)-{\hat{Y}}_{t}\left(0\right)\right),$$
  where
  (5) $${\hat{Y}}_{t}\left(0\right)=\left\{\begin{array}{cc} Y_{t} & if\;t\in T_{j}^{0}\\ \frac{1}{\#\zeta_{t}}\sum_{l\in \zeta_{t}}Y_{l} & if\;t\in T_{j}^{1} \end{array}\right.,$$
  (6) $${\hat{Y}}_{t}\left(1\right)=\left\{\begin{array}{cc} \frac{1}{\#\zeta_{t}}\sum_{l\in \zeta_{t}}Y_{l} & if\;t\in T_{j}^{0}\\ Y_{t} & if\;t\in T_{j}^{1} \end{array}\right.,$$
  $\zeta_{t}$ denotes the set of indices for the matches to unit *t*, and the number of elements of $\zeta_{t}$ is denoted by $\#\zeta_{t}$.
- **Stratification estimator:**
  (7) $${\hat{ATE}}_{Str}^{j}=\sum_{b=1}^{B_{j}}q^{b}\left({\bar{Y}}^{b}\left(1\right)-{\bar{Y}}^{b}\left(0\right)\right),$$
  where
  (8) $${\bar{Y}}^{b}\left(0\right)=\frac{1}{\#T^{b_{0}}}\sum_{t\in T^{b_{0}}}Y_{t},$$
  (9) $${\bar{Y}}^{b}\left(1\right)=\frac{1}{\#T^{b_{1}}}\sum_{t\in T^{b_{1}}}Y_{t},$$
  $q^{b}$ is the weight of the *b*th strata, $T^{b_{0}}$ and $T^{b_{1}}$ denote the set of indices for units in control and trial groups of the *b*th strata, and $B_{j}$ is the number of strata in the *j*th treatment interval.

#### 3.2.2. Integrated to the Overall Causal Effect

Based on the aforementioned process, we successfully determined the causal effect ${\hat{ATE}}^{j}$ within each treatment interval *j*. This subsection provides information on integration and obtaining the overall causal effect.

To facilitate the subsequent illustration of the integration process, we introduce a concept called ’mean difference’ in Definition 2.

**Definition** **2.** **(*Mean difference*)**. *The mean difference is the mean values’ difference between the trial and control group in each treatment interval. For any change point $\tau_{j}$ and the corresponding treatment interval $T_{j}$, the mean difference in the treatment interval is*

(10) $$D^{j}=\frac{2}{h}\sum_{t\in T_{j}^{1}}A_{t}-\frac{2}{h}\sum_{t\in T_{j}^{0}}A_{t}.$$

The causal effect within each treatment interval, $\hat{ATE^{j}}$, only represents the change in the outcome resulting from the action of the treatment variable, meaning that it captures the information from the structural change in *A*. However, it neglects the magnitude of the change in the treatment variable itself, indicating that it overlooks the information from the numeric value of *A*.

To address this limitation, we introduce the concept of ’mean difference’ to supplement this information. The mean difference quantifies the degree and direction of the observations’ fluctuation from one distribution to another, compensating for the potential information loss incurred during the initial computations. By incorporating the mean difference, the integrated overall causal effect should be determined by both the causal effect within the identified treatment interval $ATE^{j}$ estimated by Equations (2), (4) and (7) and the corresponding mean difference $D^{j}$.

**Definition** **3.** **(*Overall causal effect*)**. *With causal effect in each treatment interval j, the overall causal effect is*

(11) $$ATE=\sum_{j=1}^{J}f\left({ATE}^{j},D^{j}\right).$$

The form of function f(.) should be decided according to the real-world application. Here, we introduce two types:

- **Weighted summation estimator:**
  (12) $${\hat{ATE}}_{ws}=\sum_{j=1}^{J}\frac{|D^{j}|}{\sum_{j}|D^{j}|}{\hat{ATE}}^{j}.$$
  The weighted summation is an appropriate method based on mean difference. Mean difference presents the fluctuation degree of the treatment. A larger mean difference implies more drastic fluctuations, which provides more information and is more valuable, which should be assigned a larger weight. Thus, the overall causal effect based on weighted summation can be estimated as Equation (12).
- **Linear least square fitting estimator:**
  (13) $${\hat{ATE}}_{ls}=\frac{\sum \left(D^{j}-\bar{D}\right)\left({\hat{ATE}}^{j}-\bar{ATE}\right)}{\sum {\left(D^{j}-\bar{D}\right)}^{2}},$$
  where $\bar{D}$ is the mean of $D^{j}$, and $\bar{ATE}$ is the mean of ${\hat{ATE}}^{j}$.
  The causal effect in the large mean difference interval is different from that in the smaller mean difference interval. The linear least square fitting can combine this differing information, and the fitting coefficient can be viewed as the change degree in the overall causal effect for each unit change in the treatment variable *A*. Furthermore, this fitting coefficient can also be considered as the overall treatment effect when the mean difference changes from 0 to 1, analogous to the trial and control group. The fitting coefficient is regarded as the overall causal effect and can be estimated as the Equation (13).

### 3.3. Summary

In the preceding subsections, we detailed the steps of the proposed method, CRQ-CPD. In this method, each customer is treated as a chronological continuous treatment variable. By calculating the overall causal effect, the estimator of the β in model (1) is obtained, which allows us to determine the harmonic responsibility of each customer.

During the application of CRQ-CPD, several key points require clarification:

(1) **Assumptions of change point existence**: One of the main steps in the proposed method, identifying the crucial treatment interval, is based on change-point detection. So, the clear and discernible change points are assumed to be possessed by each customer.
(2) **Assumptions of Rubin Causal Model**: In the computation of the causal effect within the treatment interval, the three classic ps-based methods are used, requiring that Rubin’s three key assumptions, including SUTVA, Ignorability, and Positivity, should be held (detailed illustrations are described in the Appendix A).
(3) **Details of bandwidth** *h*: The notation *h* is not only the bandwidth in the change-point detection but also the length of the identified treatment intervals. In practical application, the choice of *h* may contain the subjective judgment and considerations of specific requirements. As long as the h does not exceed the space between two change points, the results are not significantly affected. By ensuring the accurate change-point detection, a shorter *h* can reduce the computational cost and save time.

Ultimately, we encapsulate the entire procedure into a comprehensive method, known as causal relation quantification, based on change-point detection, which can be accomplished by Algorithm 1.
**Algorithm 1:** β=F(Y,X).
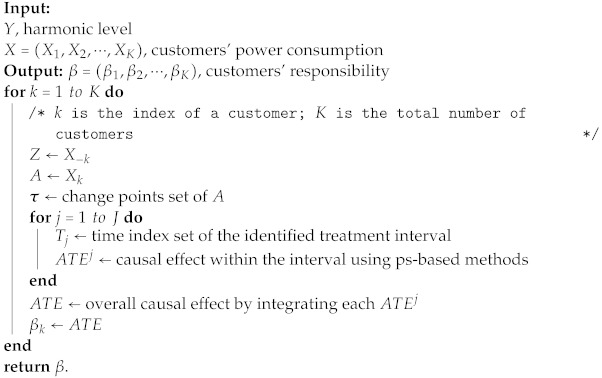


## 4. Numerical Studies

This section is composed of two parts: generating datasets to conduct simulation studies and analyzing practical responsibility division of real-world datasets. In the simulation study, ten variables are generated and used to represent customers *X*. The outcome variable *Y* is generated as the harmonic level. In order to be consistent with the goal of the actual problem, which is to obtain the responsibility of each customer rather than the fitting effect of the outcome, our evaluation of simulation results mainly focuses on the treatment’s causal effect (coefficient) on the outcome. The performance of the proposed method is examined under three scenarios: under the real model, under the real model with different signal-to-noise ratios, and under the omitted variable model. Our simulation study also examines the robustness of the proposed method. Simultaneously, in the empirical analysis, the proposed methods are employed to analyze real-world datasets with four customers, and the calculated responsibility divisions are examined according to the practical field.

### 4.1. Simulation Study

We conduct a simulation study to compare the performance of the proposed methods with existing methods: the boosting algorithm for generalized propensity score estimating (AACC) [21], generalized propensity score caliper matching (GPSMa) [22], and ordinary least squares (OLS). The vectors of customers with change points are denoted as $X=(X_{1},\cdots ,X_{10})$, where $X_{1},\cdots ,X_{4}$ are generated from $W_{1}∼N(0,1)$, $X_{5},\cdots ,X_{8}$ are generated from $W_{2}∼U(0,1)$, and $X_{9},X_{10}$ are generated from $W_{3}∼Bernoulli(0.5)$. We first generate 10 customer variables following different distributions with different means, and then set 5 mean change points for each customer variable. The change points for each customer variable were designed to not overlap with those of any other customer variables. To closely resemble real customer data, we performed a translation operation to ensure that all customer values are non-negative. This adjustment helps align the data with real-world scenarios where negative values may not be applicable or meaningful in the context of customers.

After obtaining the customer variable, the outcome variable *Y* is generated accordingly as Equation (1).

The coefficients are set as β = ($\beta_{0},\beta_{1},\cdots ,\beta_{10}$) = (3.85, 0.4, 0.42, 0, −0.36, 0.72, 0, −0.6, 0, 0.3, −0.15). The coefficient **$\beta_{k}$** (k=1,⋯,10) is assigned as the true treatment effect of each customer.

As seen, customers $X_{3}$, $X_{6}$, and $X_{8}$ are assumed to have no effect on the outcome *Y* with coefficients 0. In real-word scenarios, we are interested in whether we can identify the customers who do not cause harmonic pollution or have no responsibility. We generate 500 datasets with a sample size of 3000, and the number of change points assigned to each customer is set to 5.

To assess the quality of the estimates across simulations, we calculated the mean absolute bias (MAB) and root mean squared error (RMSE) of coefficients as

(14) $$MAB_{k}=\frac{1}{N}\sum_{i=1}^{N}\left|{\hat{\beta }}_{k}^{i}-\beta_{k}\right|,$$

(15) $$RMSE_{k}=\sqrt{\frac{1}{N}\sum_{i=1}^{N}{\left({\hat{\beta }}_{k}^{i}-\beta_{k}\right)}^{2}},$$

where ${\hat{\beta }}_{k}^{i}$ is the estimated causal effect of $X_{k}$ in the *i*th simulation, $\beta_{k}$ is the true causal effect of $X_{k}$(k=1,⋯,10 and i=1,⋯,N, with N=500).

Another important evaluation index of interest is the coefficient’s sign. If the real coefficient (causal effect) of $X_{k}$ is positive, it means that the respective customer will increases the harmonic level, while a negative coefficient of $X_{k}$ means that this customer has the ability to alleviate the harmonic level, which is beneficial. So the Signrate, defined as follows:

(16) $$Signrate_{k}=\frac{\sum_{i=1}^{N}\left[sgn\left({\hat{\beta }}_{k}^{i}\right)\cdot sgn\left(\beta_{k}\right)>0\right]}{N},$$

is used to illustrate whether these methods can identify the ’normal customer’ and the ’beneficial customer’, where sgn(•) is the sign function. Note that the Signrate values of the real coefficients equal to 0 are meaningless, so we do not compare this index of these estimations (of $X_{3}$, $X_{6}$, and $X_{8}$).

We set three scenarios in the simulation: under the real model, under the real model with different signal-to-noise ratios, and under the omitted variable model. The three indices, MAB, RMSE, and Signrate, with different methods are illustrated under the real model with a fixed signal-to-noise ratio. Since the results of these indices are similar, only RMSE is provided under the model with different signal-to-noise ratios and under omitted variable model for simplicity and intuition.

#### 4.1.1. Performance of the Proposed Methods under the Real Model

Here, we assume the real model defined by Equation (1), and the model used for practical estimation is correctly specified. As mentioned above, the main idea of this paper is to identify informative data points and partition data intervals based on the nature of change point. In the simulation, in order to enrich the experimental content and illustrate the impact of change-point detection precision on the final result, three detection methods from different aspects are selected from recent studies. Specifically, the selected methods are as follows:

(1) BOCPD (Bayesian Online Change-Point Detection) [23] is designed for online change-point detection and utilizes a message-passing algorithm to learn a probability distribution over the run length. It provides a flexible and adaptive approach for detecting change points in streaming data.
(2) DeCAFS (Detecting Changes in Autocorrelated and Fluctuating Signals) [24] is suitable for scenarios with autocorrelated noise or local mean fluctuations between abrupt changes. It focuses on capturing changes in the presence of complex noise patterns.
(3) ED-PELT (Empirical Distribution Pruned Exact Linear Time) [25] is a nonparametric change-point detection method that identifies change points by minimizing a penalty function to partition intervals. It offers a computationally efficient approach to detecting change points.

The bandwidth *h* is selected as 10logN (*N* is the length of the customer) following the SaRa method [26] and fixed in all three detection methods to facilitate comparison.

The impact of change-point detection errors can be categorized into two types: (1) false negatives, where real change points are missed, and (2) false positives, where nonchange points are falsely identified as change points.

In the first case, if there are other change points of one customer correctly identified, the final results will not be significantly influenced. Our method aims to identify major behavioral changes of the customer by detecting change points and comparing the overall harmonic levels before and after the changes. Multiple change points mean that the customer has multiple behavior changes at the entire point in time. Even if not all the behavior changes are identified, the detected change points (behavior changes) provide sufficient information on how the customer’s behavioral changes affect the overall harmonic levels, enabling the division of this customer’s harmonic responsibility.

In the second case, if a false positive change point (which is not an actual change point) is not shared with other customers’ change-point locations, the final results will not be significantly affected. In this scenario, the customer does not exhibit behavioral changes, and the overall harmonic levels also remain unchanged, aligning with the actual scenario and avoiding errors. However, if the identified positions correspond to change points of other customers, there may be significant errors in the final results. This is because the focused customer did not undergo any behavioral changes, yet the overall harmonic level changed. As a result, incorrect conclusions that even minor variations in this customer’s behavior can result in substantial changes in harmonic levels may be drawn, mistakenly magnifying this customer’s influence and inaccurately estimating its harmonic responsibility.

However, the existing methods can detect the change points precisely as shown in Table 1.

The precision of the change-point estimation is measured by the proportion of the correct number of change points in the estimated change-points set to the total number of the estimated change-points set. Let RCP denote the real change-points set, and ECP denote the estimated change-points set. The precision of the change-point estimation is defined as:

$$\begin{array}{c} \frac{\#(RCP\cap ECP)}{\#ECP}. \end{array}$$

**Figure 3:**
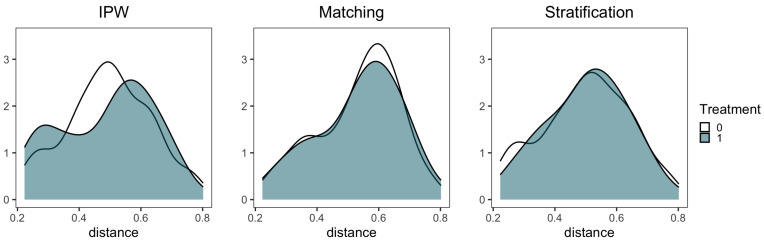
Distribution balance of a treatment interval in trial and control group of propensity score after three techniques adjusted. (This is an example where $X_{4}$ is taken as the treatment, and the other customers are taken as the covariates $Z_{1}$ to $Z_{9}$).

After accurately detecting the change points, the three classic ps-based algorithms are used to adjust the balance of the covariates. Figure 3 illustrates that the covariates follow similar distributions in the trial and control groups, indicating the three techniques based on propensity score exhibit no obvious differences to each other (an example of one treatment interval).

After balancing the covariate distributions, the causal effect within a treatment interval is calculated. For a comparison with other methods, we choose linear least square fitting here to generate the final fixed causal effect of each customer. The results are given as follows:

First, we calculate the RMSE results with IPW (Matching and Stratification are shown in the Appendix B) under the three change-point detection methods mentioned above. Table 2 illustrates that the RMSE using DeCAFS is slightly lower than that of the other methods, which is consistent with the precision results in Table 1. So the DeCAFS is used in the following computations.

**Figure 4:**
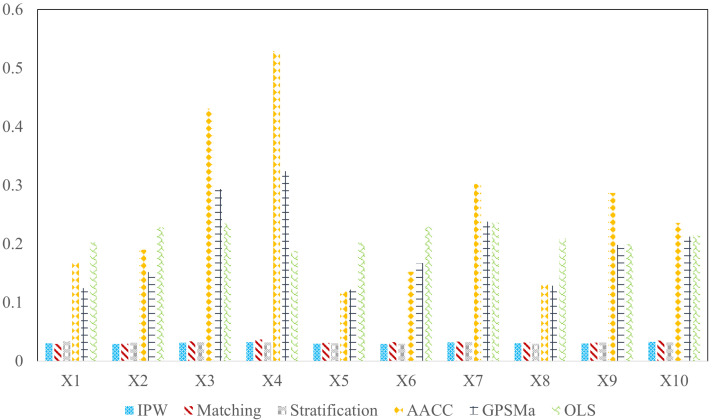
Mean absolute bias (MAB) of method comparisons.

Second, using DeCAFS, the comparing results of methods are given in Table 3 and Figure 4. It can be seen that the three proposed methods produce more accurate results than other methods, while they do not differ from each other greatly. Besides, they are not affected by variables following different distributions.

Third, the Signrate mentioned above is compared, and the results are shown in the Table 4. It can be seen that the three proposed methods provide better identification of different signs of coefficients, while AACC and GPSMa have some difficulty identifying the ’beneficial harmonic source’ as $X_{4}$ with negative coefficients. The OLS performs well when the coefficient is further away from 0 but has some difficulty identifying the coefficient near 0 as $X_{10}$.

#### 4.1.2. Performance under the Real Model with Different Signal-to-Noise Ratio

The model mentioned in the above subsection is based on the fixed signal-to-noise ratio (SNR), but in real-world applications, SNR may be different because the data quality cannot be guaranteed. A method’s insensitivity to SNR is important; so in this subsection, we discuss the methods’ robustness by setting different SNRs.

SNR is initially defined as the ratio of signal power to noise power in engineering. In statistics, SNR is used to quantify a feature of a model where an observable quantity *Y* is decomposed into a predictable or structural component Sig, often called signal or model, and a stochastic component ϵ, called noise or error [27]. In our model, it can be represented as

(17) $$\begin{array}{c} Y_{t}=\underset{Sig:\;\mathrm{predictable}\;\mathrm{component}}{\underset{︸}{\beta_{0}+\beta_{1}X_{1}+\beta_{2}X_{2}+\cdots +\beta_{10}X_{10}}}+\epsilon_{t}. \end{array}$$

As there are different definitions of SNR in different areas [28,29], we define it as follows:

(18) $$SNR=\frac{Var(Sig)}{Var(\epsilon )}.$$

We set different SNR values to test the proposed methods, and the performance is illustrated in Table 5 (for intuition and conciseness, we only provide the RMSE results).

As shown in Table 5, with the increase in SNR, the data quality is improved, so the performance of almost all of these methods becomes better. Besides, the proposed methods perform well with different SNRs and show no obvious differences, implying they are robust to noise. OLS performs the best at some variable *X* when the SNR is 100, but its RMSE increases as SNR decreases, indicating that its performance is sensitive to SNR and quite correlated to the data quality.

#### 4.1.3. Performance of the Proposed Methods under the Omitted Variable Model

In the last two subsections, we assumed that the model was correctly specified. In practicality, this may not always be true because some customers (e.g., background harmonics) that also cause harmonic pollution cannot be observed. In this case, the calculated responsibility’s accuracy of observed customers may be influenced. So, in this subsection, the models with omitted variables are considered to verify the robustness of the proposed methods. The SNR is set to 5, and the DeCAFS is used to detect change points.

Assuming that the real model is defined by Equation (1), and the models used to practically estimate are defined as M1 ($X_{5}$ omitted) and M2 ($X_{5}$ and $X_{9}$ omitted):

(19) $$\begin{array}{c} Y=\beta_{0}+\beta_{1}X_{1}+\cdots +\beta_{4}X_{4}+\beta_{6}X_{6}+\cdots +\beta_{10}X_{10}+\epsilon , \end{array}$$

where $X_{5}$ is assumed to be the omitted variable;

(20) $$\begin{array}{c} Y=\beta_{0}+\beta_{1}X_{1}+\cdots +\beta_{4}X_{4}+\beta_{6}X_{6}+\cdots +\beta_{8}X_{8}+\beta_{10}X_{10}+\epsilon , \end{array}$$

where $X_{5}$ and $X_{9}$ are assumed to be the omitted variables.

The performance of comparing methods is shown in Table 6. It can be seen that there is hardly any difference in the proposed methods’ results with or without the presence of the omitted variables, while the RMSE of the variable $X_{6}$ estimated by AACC, GPSMa, and OLS is obviously affected by the omitted variable. By comparison, the proposed methods are robust to the omitted variables.

#### 4.1.4. Robustness Analysis of the Proposed Method

In the previous three subsections, we compared the results of the method under three different scenarios. In this subsection, we investigate the robustness of the method to different data distributions and varying numbers of change points. Additionally, we present the standard error, obtained from the variance of 500 simulation estimates, and 95% confidence interval, derived from the 2.5th and 97.5th percentiles of 500 simulation estimates. The model is presented as follows:

(21) $$Y=3.85+0.4X_{1}-0.2X_{2}+0.3X_{4}+0.7X_{5}-0.15X_{6}+\epsilon$$

The customer variables $X_{1}$ and $X_{2}$ are generated from a Gaussian N(0,1) distribution, $X_{3}$ and $X_{4}$ are generated from a Student’s t(5) distribution, and $X_{5}$ and $X_{6}$ are generated from an F(5,10) distribution. The number of change points is set as 5 and 10 for each customer variable in two cases, respectively.

**Figure 5:**
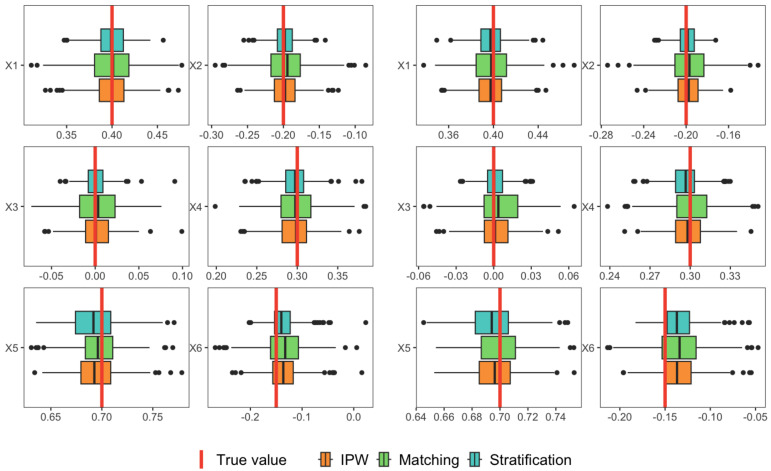
Boxplots of the proposed estimates under three data distributions (each row) with different change-point numbers (5 in **left** panel and 10 in **right** panel).

Figure 5 displays the boxplots of the proposed estimates under three data distributions (each row) with a varying number of change points. Additionally, Table 7 reports the mean, standard error (SE), and 95% confidence interval (CI) of the estimates. Comparing the results under the normal distribution and *t*-distribution, we observed the method’s robustness to heavy-tailed distributions. Similarly, comparing the results under normal distribution and *F*-distribution, we find that the method is robust to asymmetric distributions. Furthermore, the method demonstrates consistency and robustness when the number of change points varies, as evidenced by the similar results obtained for cases with 5 and 10 change points.

Overall, through comprehensive simulations, the proposed methods exhibit excellent performance in different distribution treatments with time order, providing an accurate and robust measurement for quantifying the causal relationship in responsibility division.

### 4.2. Empirical Study

In this section, the proposed methods are performed on an actual monitoring dataset to obtain the harmonic responsibility division. The actual monitoring dataset was collected from the 220 kV substation in Zhangzhou, Fujian Province, China. This dataset includes the harmonic voltage (denoted as outcome variable) in the power-quality monitoring system and the average active power data of four customers (denoted as customer variable), which were collected in the power consumption information acquisition system.

**Figure 6:**
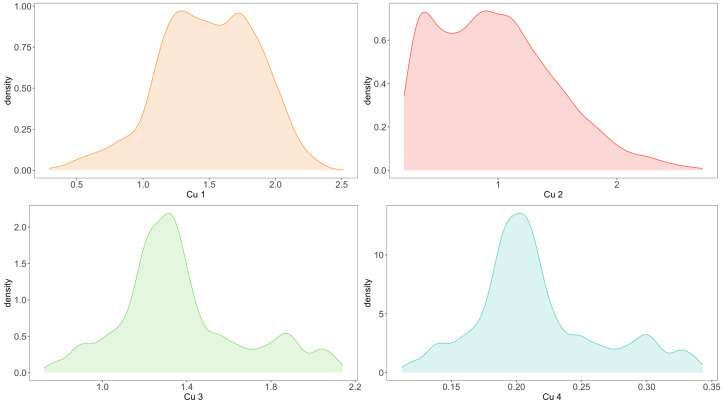
Data distribution of 4 customers.

The primary objective of our study is to assess the harmonic responsibility of customers by identifying change points in the data. Changes in customers’ power consumption behavior, represented as change points in the data, indicate a transition from one distribution to another. The focus of our analysis is on the change in behavior itself, and the specific behavior of the customer before and after the change, or the distribution of the data, is not directly relevant to the calculation of the harmonic responsibility. Regarding the empirical analysis, the distributions of the four customer variables are depicted in Figure 6.

Considering the presence of serially correlated noise, we employ the DeCAFS method to detect the change point of sequences. We identified 49 change points for Customer 1, 5 change points for Customer 2, 181 change points for Customer 3, and 30 change points for Customer 4. Given the nature of the data, a multivariate model is considered, with the regression model utilized being the usual regression model (1).

After change-point detection, some crucial treatment intervals are identified. Based on change-point detection and the three algorithms based on propensity scores, the covariates’ balance in each crucial treatment interval is achieved. Under the covariates’ balance, the treatment effect in each interval is computed. After using linear least square fitting to integrate the treatment effect in each interval, the overall causal effect, assigned as the harmonic responsibility of the customer of the chronological continuous treatment, is obtained.

In Table 8, Index is the fixed causal effect, referred to as the responsibility of each customer; Ratio is each customers’ responsibility index as a proportion of the summation of all four customers’ Index scaled by min–max standardization; and Rank represents the rank of responsibility of each customer. To account for the chronological order of the customers’ power consumption, the block bootstrap is used to compute the standard error and 95% confidence interval of the responsibility indices mentioned above. The data used for analysis is over 7 days. To preserve the structure of the time series, we divided the data into eight blocks within a block size of one day and conducted bootstrapping accordingly.

As shown in Table 8, the proposed three methods exhibit similar results. Although there is little difference between Stratification and other methods, the three methods all identify that Customer (Cu) 2 is regarded as the largest contributor of harmonic responsibility and has the most impact on the harmonic voltage. Through on-site investigation and actual measurements, the main load of Cu 2 (Manan II Road) is identified as the electric railway, which results in a large amount of harmonic emission. The remaining three loads are mainly residential electricity or small-scale industries, leading to relatively smaller harmonic emissions, which is consistent with the proposed methods.

## 5. Concluding Remarks

In this paper, we proposed the CRQ-CPD method to address the problem of harmonic responsibility division in power systems by estimating the causal effect of chronological continuous treatment. By utilizing change-point detection, we identified crucial treatment intervals and captured the main features of the treatment variable, leading to enhanced data utilization efficiency, partial preservation of chronological order, and well-adjusted covariate balance.

Within each identified interval, classic propensity score algorithms were applied to compute the causal effect. We introduced integrated methods based on mean difference to generate the overall causal effect, which serves as the measure of harmonic responsibility.

Through simulations, our proposed method demonstrated accurate results in estimating causal responsibility and effectively identified ’beneficial customers’, aiding the implementation of penalties to curb harmonic-polluting behavior in real power grids. The method showed robustness to different signal-to-noise ratios and models with omitted variables. Furthermore, empirical studies confirmed the consistency of our calculated results with on-the-spot investigations.

While our proposed method exhibited competitive performance, it does have some limitations. The accuracy of change-point detection impacts the method’s performance, particularly when there are few detected change points, leading to limited valid information for accurate estimation. Additionally, assigning harmonic responsibility to customers with overlapping change points poses challenges. Moreover, the assumption of a fixed overall causal effect at all time points should be extended to accommodate time-varying situations.

## Figures and Tables

**Table 1:** Precision of three change-point detection methods.

	BOCPD	DeCAFS	ED-PELT
$X_{1}$	95.26%	99.72%	98.00%
$X_{2}$	94.75%	99.76%	97.59%
$X_{3}$	94.34%	99.80%	97.87%
$X_{4}$	94.86%	99.88%	97.59%
$X_{5}$	98.10%	100.00%	98.31%
$X_{6}$	98.17%	100.00%	97.99%
$X_{7}$	97.68%	100.00%	98.13%
$X_{8}$	98.13%	100.00%	98.62%
$X_{9}$	97.90%	100.00%	98.72%
$X_{10}$	97.97%	100.00%	99.03%

**Table 2:** RMSE with IPW under three change-point detection methods.

	OBCPD	DeCAFS	ED-PELT
$X_{1}$	0.0555	0.0382	0.0383
$X_{2}$	0.0376	0.0368	0.0364
$X_{3}$	0.0403	0.0393	0.0393
$X_{4}$	0.0426	0.0402	0.0404
$X_{5}$	0.0379	0.0374	0.0382
$X_{6}$	0.0366	0.0372	0.0371
$X_{7}$	0.0401	0.0393	0.0395
$X_{8}$	0.0388	0.0393	0.0386
$X_{9}$	0.0386	0.0384	0.0385
$X_{10}$	0.0417	0.0409	0.0411

**Table 3:** RMSE of method comparisons.

	IPW	Matching	Stratification	AACC	GPSMa	OLS
$X_{1}$	0.0382	0.0366	0.0421	0.1927	0.1569	0.2520
$X_{2}$	0.0368	0.0377	0.0397	0.2140	0.1871	0.2792
$X_{3}$	0.0393	0.0421	0.0407	0.4448	0.3304	0.2891
$X_{4}$	0.0402	0.0457	0.0395	0.5398	0.3558	0.2335
$X_{5}$	0.0374	0.0393	0.0381	0.1469	0.1554	0.2494
$X_{6}$	0.0372	0.0397	0.0371	0.1911	0.2084	0.2817
$X_{7}$	0.0393	0.0420	0.0412	0.3309	0.2783	0.2880
$X_{8}$	0.0393	0.0405	0.0379	0.1610	0.1598	0.2545
$X_{9}$	0.0384	0.0399	0.0399	0.3129	0.2341	0.2514
$X_{10}$	0.0409	0.0433	0.0409	0.2695	0.2508	0.2607

**Table 4:** Signrate of method comparisons.

	IPW	Matching	Stratification	AACC	GPSMa	OLS
$X_{1}$	100.00%	100.00%	100.00%	100.00%	100.00%	99.40%
$X_{2}$	100.00%	100.00%	100.00%	100.00%	100.00%	94.00%
$X_{4}$	100.00%	100.00%	100.00%	63.80%	7.00%	98.00%
$X_{5}$	100.00%	100.00%	100.00%	100.00%	100.00%	100.00%
$X_{7}$	100.00%	100.00%	100.00%	98.80%	98.60%	99.00%
$X_{9}$	100.00%	100.00%	100.00%	79.20%	56.00%	88.60%
$X_{10}$	100.00%	100.00%	100.00%	97.60%	100.00%	51.20%

**Table 5:** RMSE of method comparisons with different SNR.

	SNR	IPW	Matching	Stratification	AACC	GPSMa	OLS
$X_{1}$	SNR = 5	0.0164	0.0172	0.0175	0.1202	0.0803	0.0542
	SNR = 10	0.0147	0.0165	0.0163	0.1155	0.0717	0.0368
	SNR = 100	0.0145	0.0160	0.0153	0.1080	0.0656	0.0113
$X_{2}$	SNR = 5	0.0158	0.0175	0.0180	0.1563	0.1123	0.0600
	SNR = 10	0.0153	0.0166	0.0171	0.1525	0.1083	0.0408
	SNR = 100	0.0140	0.0157	0.0163	0.1473	0.1049	0.0126
$X_{3}$	SNR = 5	0.0170	0.0245	0.0127	0.3841	0.2647	0.0622
	SNR = 10	0.0154	0.0238	0.0109	0.3794	0.2563	0.0422
	SNR = 100	0.0151	0.0232	0.0093	0.3728	0.2572	0.0130
$X_{4}$	SNR = 5	0.0224	0.0324	0.0180	0.4966	0.3313	0.0502
	SNR = 10	0.0218	0.0320	0.0171	0.4932	0.3170	0.0341
	SNR = 100	0.0213	0.0316	0.0163	0.4889	0.3252	0.0105
$X_{5}$	SNR = 5	0.0167	0.0227	0.0138	0.0867	0.0750	0.0536
	SNR = 10	0.0154	0.0220	0.0126	0.0879	0.0778	0.0364
	SNR = 100	0.0145	0.0214	0.0117	0.0921	0.0767	0.0112
$X_{6}$	SNR = 5	0.0157	0.0237	0.0116	0.0949	0.1361	0.0606
	SNR = 10	0.0146	0.0233	0.0101	0.0884	0.1312	0.0411
	SNR = 100	0.0143	0.0232	0.0089	0.0802	0.1239	0.0127
$X_{7}$	SNR = 5	0.0173	0.0243	0.0138	0.2527	0.1981	0.0619
	SNR = 10	0.0161	0.0236	0.0122	0.2468	0.1929	0.0421
	SNR = 100	0.0152	0.0229	0.0107	0.2392	0.1864	0.0130
$X_{8}$	SNR = 5	0.0161	0.0237	0.0118	0.1579	0.1362	0.0547
	SNR = 10	0.0150	0.0232	0.0104	0.1615	0.1357	0.0372
	SNR = 100	0.0139	0.0227	0.0093	0.1676	0.1420	0.0115
$X_{9}$	SNR = 5	0.0162	0.0231	0.0125	0.3338	0.2234	0.0540
	SNR = 10	0.0153	0.0225	0.0109	0.3370	0.2307	0.0367
	SNR = 100	0.0146	0.0220	0.0096	0.3422	0.2338	0.0113
$X_{10}$	SNR = 5	0.0167	0.0242	0.0131	0.3001	0.2645	0.0561
	SNR = 10	0.0153	0.0235	0.0114	0.3048	0.2670	0.0381
	SNR = 100	0.0148	0.0229	0.0099	0.3126	0.2742	0.0117

**Table 6:** RMSE of method comparisons in omitted variable models.

	Model	IPW	Matching	Stratification	AACC	GPSMa	OLS
$X_{1}$	M1	0.0185	0.0172	0.0204	0.1155	0.0713	0.0794
	M2	0.0191	0.0172	0.0201	0.1153	0.0705	0.0815
$X_{2}$	M1	0.0184	0.0175	0.0203	0.1564	0.1133	0.0727
	M2	0.0194	0.0175	0.0213	0.1564	0.1127	0.0743
$X_{3}$	M1	0.0194	0.0245	0.0166	0.3842	0.2648	0.0844
	M2	0.0201	0.0245	0.0167	0.3841	0.2663	0.0865
$X_{4}$	M1	0.0242	0.0324	0.0217	0.4654	0.3290	0.2205
	M2	0.0245	0.0324	0.0218	0.4629	0.3354	0.2240
$X_{6}$	M1	0.0181	0.0237	0.0160	0.4521	0.6043	0.3979
	M2	0.0183	0.0237	0.0168	0.4560	0.6019	0.3974
$X_{7}$	M1	0.0192	0.0243	0.0176	0.2564	0.2114	0.1008
	M2	0.0196	0.0243	0.0183	0.2561	0.2129	0.1159
$X_{8}$	M1	0.0187	0.0237	0.0172	0.1529	0.1186	0.0680
	M2	0.0185	0.0237	0.0172	0.1668	0.1947	0.1359
$X_{9}$	M1	0.0180	0.0231	0.0176	0.3336	0.2287	0.0661
$X_{10}$	M1	0.0191	0.0242	0.0160	0.3013	0.2765	0.0768
	M2	0.0193	0.0242	0.0173	0.1286	0.0660	0.1179

**Table 7:** Mean, standard error (SE), and 95% confidence interval (CI) of the proposed estimates under three distributions with 5 and 10 change points.

		IPW					Matching				Stratification			
		True Value	Mean	SE	CI		Mean	SE	CI		Mean	SE	CI	
		5 Change Points												
*N*(0,1)	$X_{1}$	0.4	0.4001	0.0219	(0.3539,	0.4424)	0.4006	0.0283	(0.3467,	0.4570)	0.3998	0.0174	(0.3669,	0.4323)
	$X_{2}$	−0.2	−0.1974	0.0224	(−0.2404,	−0.1521)	−0.1964	0.0322	(−0.2610,	−0.1382)	−0.1986	0.0161	(−0.2321,	−0.1692)
*t(5)*	$X_{3}$	0	0.0012	0.0209	(−0.0435,	0.0388)	0.0033	0.0278	(−0.0495,	0.0544)	0.0007	0.0139	(−0.0248,	0.0273)
	$X_{4}$	0.3	0.2973	0.0226	(0.2531,	0.3386)	0.2984	0.0280	(0.2474,	0.3547)	0.2970	0.0177	(0.2658,	0.3319)
*F(5,10)*	$X_{5}$	0.7	0.6945	0.0224	(0.6545,	0.7396)	0.6971	0.0214	(0.6540,	0.7389)	0.6925	0.0250	(0.6477,	0.7432)
	$X_{6}$	−0.15	−0.1365	0.0321	(−0.2037,	−0.0727)	−0.1341	0.0406	(−0.2133,	−0.0611)	−0.1378	0.0258	(−0.1860,	−0.0839)
		10 Change Points												
*N(0,1)*	$X_{1}$	0.4	0.3976	0.0155	(0.3666,	0.4281)	0.3978	0.0198	(0.3602,	0.4368)	0.3973	0.0130	(0.3727,	0.4239)
	$X_{2}$	−0.2	−0.1980	0.0139	(−0.2274,	−0.1717)	−0.1970	0.0211	(−0.2409,	−0.1575)	−0.1991	0.0098	(−0.2190,	−0.1817)
*t(5)*	$X_{3}$	0	0.0020	0.0147	(−0.0265,	0.0304)	0.0045	0.0202	(−0.0353,	0.0425)	0.0009	0.0093	(−0.0173,	0.0194)
	$X_{4}$	0.3	0.2984	0.0138	(0.2718,	0.3264)	0.3008	0.0183	(0.2621,	0.3378)	0.2965	0.0118	(0.2740,	0.3222)
*F(5,10)*	$X_{5}$	0.7	0.6963	0.0167	(0.6632,	0.7310)	0.6992	0.0167	(0.6663,	0.7304)	0.6944	0.0176	(0.6612,	0.7286)
	$X_{6}$	−0.15	−0.1346	0.0226	(−0.1768,	−0.0891)	−0.1343	0.0278	(−0.1877,	−0.0796)	−0.1347	0.0199	(−0.1683,	−0.0917)

**Table 8:** Estimated harmonic responsibility of 4 customers.

	Index				Ratio				Rank
	Estimated	SE	CI		Estimated	SE	CI		Estimated
IPW									
Cu1	0.1707	0.0562	(−0.0178,	0.1921)	29.39%	9.00%	(0.90%,	42.64%)	2
Cu2	0.3961	0.4324	(−0.8913,	0.6560)	47.87%	23.72%	(0.00%,	77.74%)	1
Cu3	0.0894	0.0208	(0.0466,	0.1274)	22.73%	8.29%	(8.71%,	44.28%)	3
Cu4	−0.1879	0.1550	(−0.3908,	0.2189)	0.00%	12.14%	(0%,	36.86%)	4
Matching									
Cu1	0.1809	0.0561	(−0.0094,	0.1986)	29.26%	9.70%	(0%,	43.71%)	2
Cu2	0.4124	0.5150	(−1.0695,	0.7322)	50.88%	24.77%	(0%,	80.02%)	**1**
Cu3	0.0803	0.0192	(0.0401,	0.1153)	19.86%	8.99%	(1.10%,	41.36%)	3
Cu4	−0.1323	0.1544	(−0.3219,	0.2817)	0.00%	12.73%	(0%,	38.34%)	4
Stratification									
Cu1	0.0413	0.0593	(−0.0663,	0.1640)	0.00%	12.17%	(0%,	40.53%)	4
Cu2	0.4600	0.5210	(−1.0114,	0.7762)	83.39%	26.17%	(0%,	82.07%)	1
Cu3	0.0986	0.0247	(0.0258,	0.1152)	11.42%	10.80%	(0%,	42.64%)	2
Cu4	0.0674	0.1954	(−0.3231,	0.4014)	5.19%	11.32%	(0%,	44.84%)	3

## Data Availability

Not applicable.

## Appendix A. Assumptions of Rubin Causal Model

**Assumption** **A1** (*Stable* *Unit* *Treatment* *Value Assumption,* *SUTVA*)**.**
*The potential outcome observation of one unit should be unaffected by the particular assignment of treatments to the other units.*

**Assumption** **A2** (*Ignorability*)**.**
*Conditional on observable covariates Z, the assignment of units to a trial or control group is independent of potential outcomes.*

(A1)
A⫫(Y(1),Y(0))|Z.

**Assumption** **A3** (*Positivity*)**.**
*The probability of receiving every value of treatment conditional on some measured covariate Z is greater than zero.*

(A2)
Pr(A=a|Z=z)>0,∀aandz.

## Appendix B. Additional Results

**Table A1:** RMSE with Matching under the three change-point detection methods.

	BOCPD	DeCAFS	ED-PELT
$X_{1}$	0.0446	0.0366	0.0369
$X_{2}$	0.0391	0.0377	0.0378
$X_{3}$	0.0433	0.0421	0.0425
$X_{4}$	0.0485	0.0457	0.0464
$X_{5}$	0.0398	0.0393	0.0397
$X_{6}$	0.0399	0.0397	0.0404
$X_{7}$	0.0423	0.0420	0.0420
$X_{8}$	0.0410	0.0405	0.0404
$X_{9}$	0.0410	0.0399	0.0408
$X_{10}$	0.0438	0.0433	0.0436

**Table A2:** RMSE with Stratification under the three change-point detection methods.

	BOCPD	DeCAFS	ED-PELT
$X_{1}$	0.0568	0.0421	0.0415
$X_{2}$	0.0416	0.0397	0.0398
$X_{3}$	0.0394	0.0407	0.0400
$X_{4}$	0.0407	0.0395	0.0394
$X_{5}$	0.0395	0.0381	0.0387
$X_{6}$	0.0384	0.0371	0.0375
$X_{7}$	0.0423	0.0412	0.0415
$X_{8}$	0.0382	0.0379	0.0384
$X_{9}$	0.0409	0.0399	0.0405
$X_{10}$	0.0402	0.0409	0.0407
